# Legal regulations of restrictions of air pollution made by non-road mobile machinery—the case study for Europe: a review

**DOI:** 10.1007/s11356-017-0847-8

**Published:** 2017-12-13

**Authors:** Konrad J. Waluś, Łukasz Warguła, Piotr Krawiec, Jarosław M. Adamiec

**Affiliations:** Faculty of Machines and Transport, Poznan University of Technology, Poznan, Poland

**Keywords:** Air pollution, Exhaust emissions, European homologation directives, Non-road mobile machinery, Combustion engine

## Abstract

The high awareness of intensification and frequency of smog phenomenon all over the world in XXI age makes for detailed analyses of the reasons of its formation and prevention. The governments of the developed countries and conscious of real hazards, including many European countries, aim to restrict the emission of harmful gases. In literature, we can find the discussions on the influence of this phenomenon on the health and life of inhabitants of contaminated areas. Some elaborations of prognostic models, descriptions of pollution sources, the manner of their restriction, and the analysis of causal-consecutive correlation are also popular. The influence of pollutions resulting from the operation of vehicles, planes, and the industry are well described. However, every machine and device which is driven with a combustion engine has the effect on the general level of anthropogenic pollutions. These drives are subject of different regulations limiting their emission for service conditions and applications. One of the groups of such machines described in European and American regulations is non-road mobile machinery. The aim of this paper is the presentation of the problem of weak analysis and application of engineering and technological tools for machinery drive emission, despite of many publications on hazards and problems of emission. These machines have the influence on both the increase of global contamination and the machine users. The regulations of the European Union take into consideration the generated hazards and restrict the emission of machine exhaust gases by approval tests—these regulations are continually improved, and the effects of these works are new emission limits in 2019. However, these activities seem to be liberal as opposed to limits of the emission for passenger and goods vehicles where the technological development of the construction is greater and the regulations are the most rigorous. During the analysis of the development of non-road mobile machinery in the correlation with automotive vehicles, we can indicate engineering and technological solutions which are limiting the emission of non-road mobile machinery, but which are not applied. Due to liberal regulations for this group of machinery, the producers do not apply innovative solutions which can be found in road vehicles. The paper presents the synthetic review of existing EU regulations concerning limits of the emission of harmful exhaust gases which are generated by spark-ignition combustion engines of non-road mobile machinery. The authors show the divergences between the limits of the emission of harmful exhaust gases generated by road vehicles and non-road mobile machinery (boats and railway engines are not taken into account). The authors present the directions of the development of the combustion process control and systems limiting the emission of harmful exhaust gases. High innovative automotive industry was indicated as the direction of the development for limiting the influence of the emission on the environment by non-road mobile machinery.

## Introduction

During last decades, the emission of air pollution in Europe was considerably decreased (Lackovičová et al. 2013; Lacressonnière et al. 2017). Concentrations of air pollution are still high, and we still have problems with the air quality (Guerreiro et al. 2014; Nieuwenhuijsen 2016; Squizzato et al. 2017). Considerable part of European population lives in areas (especially in large cities) where the standards of air quality are broken (Pascal et al. 2013; Beelen et al. 2014; Battista et al. 2016; Rodriguez et al. 2016). This situation is one of the reasons of dysphoria (Zijlema et al. 2016; Le Boennec and Salladarré 2017), loss of physical-motion capacity (Lichter et al. 2017), loss of health (Docker 2001; Oberdorster 2001; Vedal S 2002; Pereira Filho et al., 2008; Yang and Omaye 2009, Rückerl et al. 2011; Byeong-Jae et al. 2014; Sack and Kaufman 2016; Sanidas et al. 2017), and premature death (Hoek et al. 2013; WHO Regional Office for Europe and OECD 2015; Badyda et al. 2017). The sources of air pollution can be divided into natural (Liora et al. 2016) and anthropogenic ones (Avila and Alarcon 2003). Natural sources of air pollution are generated during process of e.g., chemical weathering of rocks, fire of forests and steppes, volcanic eruptions, and generation of atmospheric aerosols. Among the anthropogenic sources, we can specify ones which are generated by human activity e.g., biomass production (Beltman et al. 2013; Cordell et al. 2016), using of road vehicles (Klæboe et al. 2008; Hulskotte et al. 2014), air transport (Masiol and Harrison 2014; Koudis et al. 2017; Yılmaz 2017), sea transport (Bencs et al. 2017), mining and extractive industry (Fugiel et al. 2017), households consumption (Büchs and Schnepf 2013; Pyka and Wierzchowski 2016), and heat and power generating plants (Bieda 2011; Dulcea and Ionel 2015). More and more higher level of air pollution in conurbations leads to the implementation of legal restrictions of the emission of harmful exhaust gases in all industry branches (Szczepanek 2009; Jaś-Nowopolska 2014)—also in branch of propulsions which are applied in non-road mobile machinery. The definition of non-road mobile machinery according to the European Union directive on the emission of harmful exhaust gases is the following (Journal of Laws from 2014, No. 0, item 588):

“non-road mobile machinery shall mean transportable industrial equipment or vehicle with or without body work, not intended for the use of passenger or goods to transport on the road, or any mobile machinery intended and adopted to move on the road or rails in which an internal combustion engine is installed i.e.,:

- compression ignition engine with the net power output equal or higher than 19 kW, but not higher than 560 kW, which operates rather with a constant rotational speed and works on the basis of self-ignition principle, or
- compression ignition engine with the net power output equal or higher than 19 kW, but not higher than 560 kW, which operates with a constant rotational speed and works on the basis of self-ignition principle, or
- spark ignition engine with the net power output equal or lower than 19 kW which works on the basis of spark ignition principle and it is supplied with petrol, or
- engine designed for driving the rail cars i.e. mobile rail vehicles which are applied to transport cargo or passengers, or
- engine designed for driving the locomotives i.e. mobile rail vehicles which are applied to move freight wagons or carriages, but without locomotive service staff, or other equipment.”

Examples of non-road mobile machinery with spark ignition engine are the following: grass mowers, chainsaws, generators, water pumps, hedge trimmers, twig choppers, snow removal machines, and devices applied in forestry (Journal of Laws from 2014, No. 0, item 588). The main incentive for elaboration of innovative constructions which restrict the emission of toxic compounds in exhaust gases is the implementation of more and more rigorous homologation rules. Liberal rules of the emission of toxic compounds in exhaust gases for non-road mobile machinery lead to the common application of fuel supply systems with carburetor. On the basis of the review of present engines for non-road mobile machinery, which was performed on January 2017, one can conclude that only 11% of spark ignition engines are equipped with the electronic injection system. The advantages of the application of the electronic injection-ignition systems are the following: lower fuel consumption, lower content of toxic compounds in exhaust gases, higher efficiency of combustion process, power and torque increase, improvement of reaction dynamics on the variation of operating conditions, reduction of number of control-service activity, general reduction of the level of impact on the environment due to self-diagnosis of system, the ability to work in emergency mode and indicator of system failure MIL (malfunction indicator lamp), facilitation of system diagnosis by the use of diagnostic functions—work parameters monitoring and failure code recording (Warguła and Waluś 2014; Waluś et al. 2017). Among the main manufacturers of combustion engines with spark ignition for non-road mobile machinery who apply the electronic injection-ignition systems EFI (electronic fuel injection), we can enumerate the following ones: Kohler Engines, Briggs & Stratton, Honda, Kawasaki, Subaru Robin, and Yamaha. Additionally, ECOTRONS company produces modification kits for such type of engines which allow to implement the electronic injection-ignition system. The applied systems are different and deviate from the standards of present produced passengers vehicles which are pioneers in the branch of combustion engine development. The solutions used in EFI systems for combustion engines of non-road mobile machinery had been also commonly applied in automotive vehicles. These ones were displaced by newer solutions or were additionally equipped with the innovative systems. In case of circuits, systems, and subassemblies, which are characteristic for present construction elements (reducing the emission of harmful gases) of the injection-ignition systems in combustion engines with spark ignition for mobile machinery propulsions, the following elements are not applied: resistance sensor of oxygen content in exhaust gases (broadband Lambda sensor) (Ivers-Tiffée et al. 2001; Riegel et al. 2002; Ramamoorthy et al. 2003; Wales et al. 2015), a few oxygen detectors in exhaust gases (Twigg 2006), nitrogen oxides detectors in exhaust gases (Ménil et al. 2000; Moos 2005), direct injection to combustion chamber (Badami et al. 1999; Zhao et al. 1999), absorption of fuel vaporous (Jentz et al. 2014), three-function catalytic reactor (Heck and Farrauto 2001; Twigg 2007), measurement of air mass flow with wire anemometer HLM (Hitzdraht luftmassenmesser) or plate anemometer HFM (Heißfilmluftmassenmesser), system of additional air blow to exhaust manifold (Sathish and Loganathan 2017), exhaust gas recirculation system (Yu et al. 2013; Ueda 2015), systems with the dynamic supercharging phenomenon—supercharging with the use of turbocompressor, mechanical compressor or Comprex compressor, start-stop system, and variable valve timing.

The emission restriction is also necessary due to its direct influence on humans’ health who live not only in Europe (Zeng et al. 2016; Tang et al. 2017).

There are conducted some research on the emission level generated by combustion engines of non-road mobile machinery and its influence on the environment and operators (Czerwinski et al., 2014; Lijewski 2015; Merkisz et al. 2016; Liu et al. 2016; Dimou et al. 2017; Hooper et al. 2017). The research were performed in China in year 2006 (Zhang et al. 2006), and these ones showed that the emission of PM2.5 from road vehicles was equal to 123,000 tons, and from the propulsions of non-road machinery was equal to 38,000 tons (Wang et al. 2016). The results of research conducted in the USA in year 2006 showed that the operators of machinery with such type of combustion engines can be endangered to the increased level of CO and PM2.5 which can be higher about two orders of magnitude (Baldauf et al. 2006). It means that the emission generated by such type of machinery cannot be ignored, because it is a part of global contamination of the world, but it is also a significant local contamination of the workstation and local health and life hazard.

In literature, one can find the subject matter of legal regulations on the emission of air pollutions generated in Europe. We can indicate the following directions of legal and political aspirations to the reduction of the emission of toxic exhaust gases from combustion engines:

- rigorous emission standards for the stage of certification, operation, and production;
- investments into the technological development of constructions which are using products made of petroleum and constructions which are independent from the petroleum;
- reforms of fuel tax.

Among all the calculations of the generated emission in Europe or in the selected European areas and all the determinations of the results of the introduced solutions for emission limits, the scientists, whose papers are described below, state that the effects of introduced solutions are ambiguous or very complicated. Bagayev and Lochard’s paper from the year 2017 concerning the adjustment of air pollutions generated by the industry in the European Union, indicates that the developed countries of EU implement more rigorous emission standards of air pollutions than the developing countries of Europe or Asia, because these ones import from those ones more products which are intensively polluting the air (Bagayev and Lochard 2017). In 2014, Rafaj showed that the limitation strategy of the emission of air pollutions TSAP (Thematic Strategy on Air Pollution), which was taken by the European Committee in 2005, will not be fulfilled without any additional activities (Rafaj et al. 2014). In 2017, Lacressonničre et al. indicated that the annual value of PM10 can be reduced about ca. 1.8–2.9 μg/m^3^ in compliance with the present legal scenario—especially for the countries of Benelux (Lacressonnière et al., 2017). When maximum technological possibilities of the emission reduction will be implemented, then the annual value of PM10 can reach the level of 1.4–1.9 μg/m^3^ (Lacressonnière et al. 2017). The global and overall determination of the emission level of pollutions for specific geographic areas is complicated and multidimensional—this issue is discussed by e.g., Ederington, Levinson and Taylor, Brunel and Levinson (Ederington et al. 2005; Levinson and Taylor 2008; Brunel and Levinson, 2016). Completely different manners limiting the emission of exhaust gases in Europe are tax reforms for the purpose of the limitation of harmful pollutions of air and the emission of the carbon dioxide in the road transport. The results of such political interventions were described by Zimmer and Koch in 2017—they indicated that the fuel consumption is elastic, and the results of reforms have significantly allowed to achieve the aims of the EU within the range of policy of the counteractions to climatic changes for the year 2020 (Zimmer and Koch 2017). The existing policy in Europe within the range of the emission limitation of the air pollution is not coherent, but the directions of the emission limitation with the technology, rigorous regulations of approval or fuel tax regulations give the measurable advantages in the form of the emission reduction (Creutzig et al. 2011). The investigations showed a strong synergy between the climatic policy and the policy within the range of air pollutions (Bollen and Brink 2014; Nam et al. 2014). The motor market for the purpose of supporting the reduction of climate changes decreases its dependence from the petroleum. In EU, the works on standards of the fuel efficiency has begun from the voluntary agreement which established the general aim to 140gCO_2_/km for EU and it must be reached by European, Japanese and Korean carmakers. In 2009, it was indicated that until the year 2015, this value has to be equal 130 g CO_2_/km (Creutzig et al. 2011). The reduction about 10 g CO_2_/km can be obtained by implementing the actions such as efficient tires, air conditioning, tire pressure monitoring, and gear shift indicators (Regulation (EC), 2009). Long-term aim for the year 2020 is equal 95 CO_2_/km (Creutzig et al. 2011). The authors of the above papers confirm Bagayev and Lochard’s conclusion from 2017 that the influence of regulations of environment protection and economy on the air quality cannot be unequivocally determined (Bagayev and Lochard 2017). The presented paper indicates that one of solutions for the human health protection can be the limitation of the emission from engines of non-road mobile machinery. In opposite to global regulations connected with the emission of devices, the homologation restrictions force the producers to make environment-friendly devices and displace devices and machines made in older technology. The obtained effects of rigorous emission standards have the unequivocal influence on the emission reduction.

The paper presents the European homologation rules for combustion engines with spark ignition for non-road mobile machinery. The rules for this type of machinery were referred to the homologation rules of the other group of combustion engines for road vehicles mainly where the allowable limits of the emission are more rigorous. This analysis does not take into consideration the combustion engines with compression ignition and the engines for boats and locomotives. The aim of this paper is to present the significant differences between the rules for combustion engines of non-road mobile machinery and automotive vehicles. The allowable European limits for emission of toxic compounds in exhaust gases—nowadays and after the implementation of the directive 2016/1628/EU in year 2019—are very liberal. It allows the manufacturers to sell the constructions with old technologies. This situation additionally does not force the manufacturers to invest in innovative constructional solutions. The present development of control technology of combustion processes and limiting systems of the emission of toxic compounds in exhaust gases which are applied in automotive industry allow to restrict considerably the influence of this type of propulsions on the environment. The automotive industry shows the direction of the propulsions development for non-road mobile machinery which allows to protect the environment in the whole world.

## The European homologation rules

In Europe, first regulations for non-road vehicles were presented in December 1997 (Directive, 1997/68/EC). The regulations for limiting the emission level of toxic compounds were implemented in two stages (Stage I and Stage II)—first stage was implemented in 1999, and second one was implemented in years 2001–2004 for different engine powers. First European Union standards concerning the emission for combustion engines with spark ignition below 19 kW applied in non-road mobile machinery were implemented in 2002.

Stages I and II were implemented by the Directive, 2002a/88/EC which has replaced the Directive, 1997/68/EC. The directive from year 2002 has implemented the standards for the emission of spark ignition engines with small power below 19 kW for non-road mobile machinery. It has also implemented the division of engines into the following classes: engines for hand-held machinery—H, and engines for non-hand-held machinery—N (Table 1).

**Table 1:** Spark ignition engines—ranked to S class (engines with a net power ≤ 19 kW) (Journal of Laws from 2014, No. 0, item 588)

Class	Displacement (cm^3^)
Hand-held engines	
SH:1	< 20
SH:2	≥ 20
	< 50
SH:3	≥ 50
Non-hand-held engines	
SN:1	< 66
SN:2	≥ 66
	< 100
SN:3	≥ 100
	< 225
SN:4	≥ 225

Emission limits for Stage I are given in Table 2, and for Stage II are given in Table 3. These standards are applied for spark ignition engines with a net power below 19 kW (class S). Irrespective of the definition of hand-held two stroke engine, in Stage I and II, the engines for mechanical snow removal machines must satisfy only the emission limits for SH:1, SH:2, or SH:3 (DieselNet- Emission Standards, 2017).

**Table 2:** Emission limits of toxic compounds in exhaust gases—Stage I in Europe for spark ignition engines with the power below 19 kW of non-road mobile machinery and date of implementation (DieselNet- Emission Standards, 2017; Directive, 1997/68/WE; Directive 2001/63/WE; Directive, 2002a/88 /WE; Directive 2004/26/WE; Directive, 2010a/26/UE; Directive 2011/88/UE; Directive 2012/46/UE)

Class	Date	Carbon monoxide (CO)	Hydrocarbons (HC)	Nitrogen oxides (NO_x_)	Sum of hydrocarbons and oxides of nitrogen (HC + NO_x_)
		(g/kWh)			
Hand-held engines					
SH:1	2004	805	295	5.36	
SH:2		805	241	5.36	
SH:3		603	161	5.36	
Non-hand-held engines					
SN:1	2004	519	–	–	50
SN:2		519	–	–	40
SN:3		519	–	–	16.1
SN:4		519	–	–	13.4

**Table 3:** Emission limits of toxic compounds in exhaust gases—Stage II in Europe for spark ignition engines with the power below 19 kW of non-road mobile machinery and date of implementation (DieselNet- Emission Standards, 2017; Directive, 199768/WE; Directive 2001/63/WE; Directive, 2002b/88 /WE; Directive 2004/26/WE; Directive, 2010b/26/UE; Directive 2011/88/UE; Directive 2012/46/UE)

Class	Date	Carbon monoxide (CO)	Hydrocarbons (HC)	Nitrogen oxides (NO_x_)	Sum of hydrocarbons and oxides of nitrogen (HC + NO_x_)*
		(g/kWh)			
Hand-held engines					
SH:1	2007	805	–	–	50
SH:2		805	–	–	50
SH:3	2008	603	–	–	72
Non-hand-held engines					
SN:1	2004	610	–	–	50
SN:2		610	–	–	40
SN:3	2007	610	–	–	16.1
SN:4	2006	610	–	–	12.1

*Additionally, NOx for all engine classes cannot exceed 10 g/kWh

Next legal document concerning the emission from non-road vehicles is the Directive 2004/26/EC from the year 2004. On the basis of this document, next emission limits of toxic compounds were implemented in two stages: III and IV (Stage III and Stage IV)—it was distributed in years 2006–2014. Additionally, Stage III was divided into two sub-stages: IIIA and IIIB. Stage IIIB incorporates very restrictive limits for the emission of nitrogen oxides (NO_x_) and particulate solids (PM)—the emission of particulate solids was reduced about 90% in relation to Stage II. In 2015, next document was elaborated and implemented, i.e., the Directive 2005/13/EC concerning the engines of vehicles applied in agriculture and forestry. In 2010, two additional directives were implemented: the Directive, 2010a/26/UE—it incorporates further technical details on testing and homologation of engines from Stage IIIB and IV; the Directive, 2010b/22/UE—it changes the earlier regulations for vehicles which are applied in agriculture and forestry. The regulations described in Stages III and IV do not concern to spark ignition combustion engines with net power below 19 kW.

In 2016, the standards of Stage V were accepted—these will be obligatory from 2019—the emission regulations were restricted and classes of spark ignition engines were enlarged. Stage V of the emission regulations intensifies the emission standards for existing engine classes and enlarges the number of engine classes for the engines which have not been taken into consideration in earlier regulations. We can distinguish category NRSh—the spark ignition engines with the power below 19 kW used in hand-held applications only (Table 4). We can also distinguish category NRS—the spark ignition engines with the power below 56 kW which have not been taken into consideration of category NRSh (Table 5). We can also distinguish category SMB—for spark ignition engines applied in snowmobiles, and category ATS—for spark ignition engines applied in “all terrain” and “side-by-side” vehicles. The engines with the power higher than 56 kW must satisfy the emission standards for engines from category NRE. This category includes all types of engines with the power higher than 56 kW both for spark and compression ignition (Regulation 2016/1628/UE). The emission limits of Stage V for spark ignition engines with the power below 19 kW applied in hand-held machinery and spark ignition engines with the power up to 56 kW applied in non-hand-held machinery are given in Table 6. Emission limits of toxic compounds in exhaust gases of Stage V in Europe for spark ignition engines applied in propulsions of snowmobiles (SMB-v-1) and “all terrain” and “side-by-side” vehicles (ATS-v-1) are presented in Table 7. Emission in Stage V is measured in different test cycles depending on the machinery service conditions (e.g., DieselNet- Emission Standards, 2017; Regulation 2016/1628/UE).

**Table 4:** Division of spark ignition engines of class S with the net power ≤ 19 kW according to Stage V, applied in hand-held machinery (DieselNet- Emission Standards, 2017; Regulation 2016/1628/UE)

Category	Displacement (cm^3^)
Hand-held engines	
NRSh-v-1a	< 50
NRSh-v-1b	≥ 50

**Table 5:** Division of spark ignition engines of class S with the net power ≤ 56 kW according to Stage V, applied in non-hand-held machinery (DieselNet- Emission Standards, 2017; Regulation 2016/1628/UE)

Category	Displacement (cm^3^)	Power (kW)	Speed operation (rpm)
Non-hand-held engines			
NRSh-vr-1a	≥ 80	< 19	Variable ≥ 3600; or constant
	< 225		
NRSh-vr-1b	≥ 255		
NRSh-vi-1a	≥ 80		Variable < 3600
	< 225		
NRSh-vi-1b	≥ 255		
NRS-v-2a	≤ 1000	≤ 19	Variable or constant
		> 30	
NRS-v-2b	> 1000	≤ 30	
NRS-v-3	Any	> 56

**Table 6:** Emission limits of toxic compounds in exhaust gases according to Stage V in Europe for spark ignition engines with the power below 19 kW applied in hand-held mobile machinery and with the power up to 56 kW applied in non-hand-held mobile machinery and date of implementation (DieselNet- Emission Standards, 2017; Regulation 2016/1628/UE)

Category	Date	Carbon monoxide (CO)	Sum of hydrocarbons and oxides of nitrogen (HC + NO_x_)*
		(g/kWh)	
Hand-held engines			
NRSh-v-1a	2019	805	50
NRSh-v-1b		603	72
Non-hand-held engines			
NRS-vr/vi-1a	2019	610	10
NRS-vr/vi-1b		610	8
NRS-v-2a		610	8
NRS-v-2b		4.40*	2.70*
NRS-v-3		4.40*	2.70*

*or any combination of the values which satisfy the equation: (HC + NO_x_)·CO^0.784^ ≤ 8.57, conditions: CO ≤ 20.6 g/kWh and (HC + NO_x_) ≤ 2.7 g/kWh

**Table 7:** Emission limits of toxic compounds in exhaust gases of Stage V in Europe for spark ignition engines applied in propulsions of snowmobiles (SMB-v-1) and “all terrain” vehicles (ATS-v-1) and data of categories implementation (DieselNet- Emission Standards, 2017; Regulation 2016/1628/UE)

Category	Date	Power	Carbon monoxide (CO)	Sum of hydrocarbons and oxides of nitrogen (HC + NO_x_)
		(kW)	(g/kWh)	
SMB-v-1	2019	> 0	275	75
ATS-v-1	2019	> 0	400	8

In next years, the legislation concerning Stage V obligates the European Commission to present two reports on further regulations of the exhaust gases emission. First report must be ready until the end of year 2018, and it will refer to estimation of possibilities to take some actions connected with the installation of additional devices for emission control in existing (used) non-road vehicles. Second report must be ready until the end of year 2020, and it will refer to estimation of further potential reduction of contamination emission and identification of potentially existing types of contaminations which are not incorporated in regulations of Stage V (DieselNet- Emission Standards, 2017).

Apart from regulations in the European Union, some additional regulations of the emission for non-road vehicles are implemented by some countries. The example of such activity is the establishment of low-emission zone LEZ—this is an area where operation of vehicles which contaminate the environment is restricted, in this area the following types of vehicles can be used: alternative fuel vehicles, hybrid electric vehicles, or zero-emission vehicles such as all-electric vehicles. In 2015, over 200 zones of such type were established in Europe (Cruz and Montenon 2016). In 1996, the largest cities in Sweden implemented the program Environmental Zones—the main aim of this program was the improvement of the air quality by the traffic restriction of HDV (Heavy-Duty Vehicles) vehicles in city centers. In 1999, the similar program was implemented, and it referred to non-road vehicles. According to the accepted project, all engines which do not satisfy the European or American standards (Stage I/Tier 1) must be equipped with a catalytic reactor when these ones achieve the age of 8, and in some cases, these ones must be equipped with a filter of solid particulates DPF (diesel particulate filter) (Merkisz and Walasik 2006). In Switzerland, similarly as in Sweden, one of the main actions is the assembly of diesel particulate filters. Initially, since year 2000, the obligation of assembly of diesel particulate filters was implemented for building machinery applied in underground constructions (tunnels and parking places), and since 2002, for all machinery applied in large constructions (Merkisz and Walasik 2006).

## The analysis of homologation research methods of the emission of non-road combustion engines with spark ignition

Homologation tests of combustion engines applied for drives of non-road mobile machinery are obligatory in the European Union countries, and these tests are orientated on the emission of toxic compounds of exhaust gases. Standard ISO 8178, elaborated by the International Standard Organization (ISO), consists of the following 10 parts:

- part 1: Test-bed measurement systems of gaseous and particulate emissions (ISO 8178-1 2017)
- part 2: Measurement of gaseous and particulate exhaust emissions under field conditions (ISO 8178-2 2008)
- part 3: Definitions and methods of measurement of exhaust gas smoke under steady-state conditions (ISO 8178-3 1994)
- part 4: Steady-state and transient test cycles for different engine applications (ISO 8178-4 2017)
- part 5: Test fuels (ISO 8178-5 2015)
- part 6: Report of measuring results and test (ISO 8178-6 2000)
- part 7: Engine family determination (ISO 8178-7 2015)
- Part 8: Engine group determination (ISO 8178-8 2015)
- part 9: Test cycles and test procedures for test bed measurement of exhaust gas smoke emissions from compression ignition engines operating under transient conditions (ISO 8178-9 2012)
- part 10: Test cycles and test procedures for field measurement of exhaust gas smoke emissions from compression ignition engines operating under transient conditions (ISO 8178-10 2002)
- part 11: Test-bed measurement of gaseous and particulate exhaust emissions from engines used in non-road mobile machinery under transient test conditions (Withdrawn in 2014.08.13) (ISO 8178-11 2006)

Test procedure can be done with two types of test cycles: NRSC—stationary cycle for non-road machinery (Non-Road Stationary Cycle), and NRTC—nonstationary cycle for non-road machinery (Non-Road Transient Cycle). Additionally, depending on the application of the device, types of service conditions or group and series of engines, the tests are performed in defined test cycles which differ in service conditions and number of research phases. On the basis of conducted tests, one can calculate the mean emission of the individual toxic compounds of exhaust gases—depending on the application of the examined engine one can match characteristic contribution coefficients for every phase of the test. Table 8 presents types of test cycles for different category groups of engines, i.e., NRSh, NRS, SMB, and ATS.

**Table 8:** NRSC test cycles for engines of category NRSh, NRS, SMB, and ATS (Journal of Laws from 2014, No. 0, item 588)

Category	NRSC
NRSh-v-1a	G3
NRSh-v-1b	
NRS-vi-1a	G1
NRS-vi-1b	
NRS-vr-1a	G2
NRS-vr-1b	
NRS-v-2a	
NRS-v-2b	C2
NRS-v-3	C2
SMB-v-1	H
ATS-v-1	G1

Test cycle of non-road mobile machinery equipped with spark ignition engines with the net power not higher than 19 kW is done for defined test cycles and depends on the application of device and its service conditions. Type of driven machine determines the following measuring cycles of the engine which is connected with the brake:

- cycle D (it is identical with cycle D2 according to standard ISO 8168-4: 1996(E)): engines with a constant rotational speed and with different loads such as in power generators;
- cycle G1: it is applied to engines mounted in machines with different rotational speed which are not hold in hands;
- cycle G2: it is applied to engines with rated rotational speed which are not hold in hands;
- cycle G3: it is applied to engines mounted in machines which are hold in hands.

Exemplary test phases and weighting factors are presented in Table 9 where the rated rotational speed is equal to maximal one at full load—it is restricted with governor according to producer’s data. The indicated intermediate rotational speed is equal to rotational speed of the engine when the following conditions are satisfied:

- for the case of engines which work in range of rotational speed on the moment curve at full load, the intermediate rotational speed should be taken as declared rotational speed of maximal moment—if this moment exists between 60 and 75% of rated rotational speed,
- if the declared rotational speed of maximal moment is less than 60% of rated rotational speed, then intermediate rotational speed should be equal to 60% of rated rotational speed,
- if the declared rotational speed of maximal moment is higher than 75% of rated rotational speed, then intermediate rotational speed should be equal to 75% of rated rotational speed,
- for the case of engines which are assigned to tests according to cycle G1, the rotational speed should be equal to 85% of rated rotational speed.

**Table 9:** Test phases and weighting factors for test cycle D, G1, G2, G3 (Journal of Laws from 2014, No. 0, item 588)

Cycle D											
Number of phase	1	2	3	4	5						
Rotational speed of engine			Rated rotational speed			Intermediate rotational speed			Rotational speed of idle running		
Load* (%)	100	75	50	25	10						
Weighting factor	0.05	0.25	0.3	0.3	0.1						
Cycle G1											
Number of phase						1	2	3	4	5	6
Rotational speed of engine			Rated rotational speed			Intermediate rotational speed			Rotational speed of idle running		
Load (%)						100	75	50	25	10	0
Weighting factor						0.09	0.2	0.29	0.3	0.07	0.05
Cycle G2											
Number of phase	1	2	3	4	5						6
Rotational speed of engine			Rated rotational speed			Intermediate rotational speed			Rotational speed of idle running		
Load (%)	100	75	50	25	10						0
Weighting factor	0.09	0.2	0.29	0.3	0.07						0.05
Cycle G3											
Number of phase	1										2
Rotational speed of engine			Rated rotational speed			Intermediate rotational speed			Rotational speed of idle running		
Load (%)	100										0
Weighting factor	0.85**										0.15**

*Load values are expressed as percentage values of torque which corresponds to the basic value of the power defined as the maximal power obtained during variable power sequence—it can be received during unlimited number of hours in the year between the defined service periods and for the given environmental conditions; maintenance is done according to producer’s data

**For Stage I, the following values can be taken 0.90 and 0.10 instead of 0.85 and 0.15

The selection of a proper test cycle, when we know the last application of engine model, can be done on the basis of the below presented examples. If the last application of engine is unknown, then test cycle should be done on the basis of technical data of the engine. Typical examples of test cycles for the following applications:

- Cycle D: generating sets with intermittent load including generating sets on board ships and trains (not for propulsion), refrigerating units, welding sets, gas compressors.
- Cycle G1: front or rear engines riding lawn mowers, golf carts, lawn sweepers, pedestrian-controlled rotary, or cylinder lawn mowers, snow-removal equipment, waste disposers.
- Cycle G2: portable generators, pumps, welders, and air compressors may also include lawn and garden equipment, which operate at engine rated speed.
- Cycle G3: blowers, chain saws, hedge trimmers, portable saw mills, rotary tillers, sprayers, string trimmers, vacuum equipment.

The valid homologation regulations of combustion engines of non-road mobile machinery during research tests allow to represent the real service conditions of the engines. The investigations performed in 2017 show that the measured emission of HC and CO generated (in real operation conditions) by selected non-road mobile machinery equipped with spark ignition engines are higher than the allowable values. This fact results from different service conditions of engine than defined ones by standards of homologation tests. Additionally, these investigations show that the applied research method and PEMS apparatus (Portable Emission Measurement System) should be implemented into the homologation procedure (Lijewski et al. 2017). The fundamental advantage of such tests is the coverage of all actual service conditions and aspects of combustion engine, which is hardly reproducible under laboratory conditions during tests performed on engine test beds.

The actual state of regulations in Europe in scope of the emission investigations of toxic compounds of exhaust gases do not impose any obligations (apart from homologation tests) of control examinations of machine exhaust gas emission for producers and users of these machines. In spite of large efforts on the design and production phase, the exhaust gases emission of the engine can be highly increased during machine service due to wear or damage of engine elements. Therefore, it is well motivated to implement control procedures which will allow to detect the increased emission of the exhaust gases of the operating engine. This requires the elaboration of research methodology and implementation of proper regulations. Additionally, in case of non-road vehicles the engine and its elements are often exposed to failures due to specific service conditions. A lack of control procedures for vehicles is the reason of uncontrolled emission of toxic compounds from engines (Merkisz et al. 2010).

The regulations implemented by the European Parliament play a decisive role during the elaboration of progressive policy in the field of environment which will be applied to fight against the air pollution in Europe. The regulations and research methods on air pollution restrictions for engines should be resistant to such situations as in case of discrepancy between the real emissions and test emissions of the vehicles with diesel engines which satisfy the emission standard Euro 6. The example of such situation is the application of software to falsify the test results of NO_x_ emission in the vehicles of the Volkswagen Group (Ohliger 2017).

## The analysis of the European homologation directives

Homologation rules for combustion engines used in road applications have been implemented in 1990s. Nowadays, the regulations relating to the emission of this group of engines are more rigorous than for the engines applied in non-road mobile machinery. This situation has an impact on design and construction of the applied supply and control systems for combustion engines. Non-road mobile machines are propelled mainly with petrol or diesel fuel. Apart from the abovementioned fuels, the manufacturers of leading makes introduce into service the engines which are supplied with alternative fuels e.g., LPG, CNG, kerosene, or biofuels. The solutions of the emission restriction for these engines are usually taken from the engines for road vehicles with few or several years delay.

**Fig. 1:**
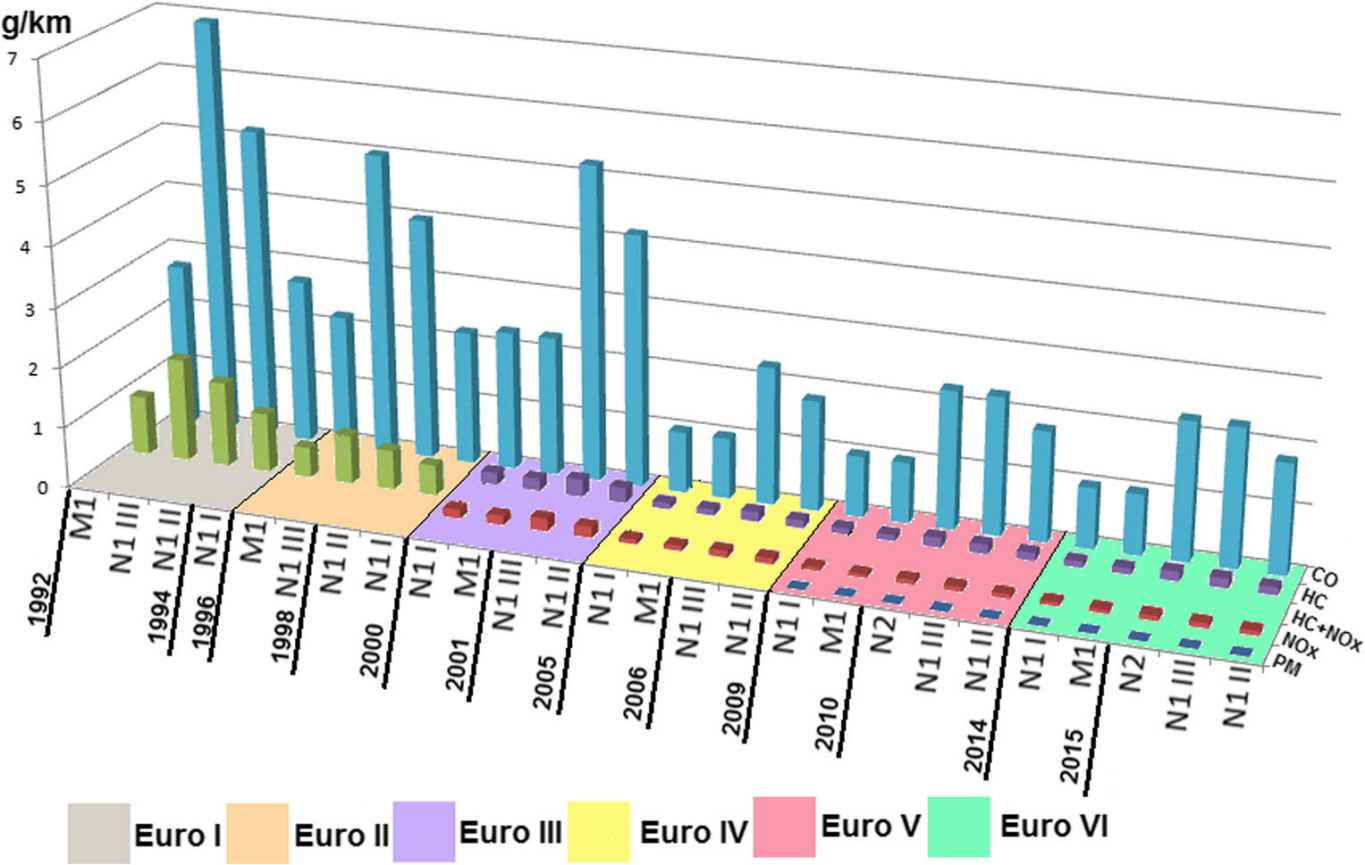
The European tendency of decreasing the emission limits of harmful exhaust gases for passenger vehicles and light delivery vehicles with spark ignition engine

The emission limits of toxic compounds in exhaust gases of Euro I-VI in Europe for spark ignition engines applied in passenger vehicles are measured in units (g/km) which does not allow to compare the implemented emission limits for non-road mobile machinery. The emission limits of passenger vehicles and light delivery vehicles classified as M1, N1, and classes I, II, III, and N2 are characterized with the most rigorous standards of exhaust gases emission (Table 11). Table 10 and Fig. 1 present a comparison of the European emission limits for passenger vehicles with up to eight seats excluding the driver’s seat (M1) and for goods vehicles with maximal load capacity lower than 3.5 tons (N1) and higher than 3.5 tones, but which does not exceed 12 tons (N2). The emission limits for passenger vehicles decrease with increasing the class or category which depends on the allowable vehicle weight.

**Table 10:** Allowable European emission limits of harmful exhaust gases for passenger vehicles and light delivery vehicles with spark ignition engine and date of implementation (Lindqvist, 2012; Gołębiowski et al. 2013; Delphi-Worldwide Emissions Standards, 2017; Council Directive, 1991, 1993; Directive, 1994/12/WE; Directive, 1996/69/WE; Directive, 1998/69/WE; Directive, 2002a/80/WE; Regulation 2007/715/WE)

Passenger vehicles (category M1*)							
Euro	Date	CO	HC	HC + NO_x_	NO_x_	PM	PN
		g/km					#/km
I	1992	2.72 (3.16)	–	0.97 (1,13)	–	–	
II	1996	2.20	–	0.5	–	–	
III	2000	2.30	0.20	–	0.15	–	
IV	2005	1.0	0.10	–	0.08	–	
V	2009^a^	1.0	0.10^b^	–	0.06	0.005^c,d^	
VI	2014	1.0	0.10^b^	–	0.06	0.005^c,d^	6.0 × 10^11c,e^
Light delivery vehicles, class I, ≤ 1305 kg (category N1)							
I	1994	2.72	–	0.97	–		
II	1998	2.2	–	0.50	–		
III	2000	2.3	0.20	–	0.15		
IV	2005	1.0	0.10	–	0.08		
V	2009^a^	1.0	0.10^b^	–	0.06	0.005^c,d^	
VI	2014	1.0	0.10^b^	–	0.06	0.005^c,d^	6.0 × 10^11c,e^
Light delivery vehicles, class II, 1305–1760 kg (category N1)							
I	1994	5.17	–	1.40	–	–	
II	1998	4.0	–	0.65	–	–	
III	2001	4.17	0.25	–	0.18	–	
IV	2006	1.81	0.13	–	0.10	–	
V	2010^g^	1.81	0.13^h^	–	0.075	0.005^c,d^	
VI	2015	1.81	0.13^h^	–	0.075	0.005^c,d^	6.0 × 10^11c,e^
Light delivery vehicles, class III, > 1760 kg (category N1)							
I	1994	6.90	–	1.70	–	–	–
II	1998	5.0	–	0.80	–	–	–
III	2001	5.22	0.29	–	0.21	–	–
IV	2006	2.27	0.16	–	0.11	–	–
V	2010^g^	2.27	0.16^h^	–	0.082	0.005^c,d^	–
VI	2015	2.27	0.16^h^	–	0.082	0.005^c,d^	6.0 × 10^11c,e^
Light delivery vehicles (category N2)							
V	2010^g^	2.27	0.16^h^	–	0.082	0.005^c,d^	–
VI	2015	2.27	0.16^h^	–	0.082	0.005^c,d^	6.0 × 10^11c,e^

*During Euro 1.4 stages, passenger vehicles > 2500 kg have been homologated as vehicles of category N_1_

^a^2011 for all models

^b^NMHC = 0.068 g/km

^c^Applicable only to vehicles using DI engines

^d^0.0045 g/km using the PMP measurement procedure

^e^6.0 × 10^12^ 1/km within first 3 years from Euro 6 effective dates

^f^NMHC = 0.090 g/km

^g^2012.01 for all models

^h^NMHC = 0.108 g/km

**Fig. 2:**
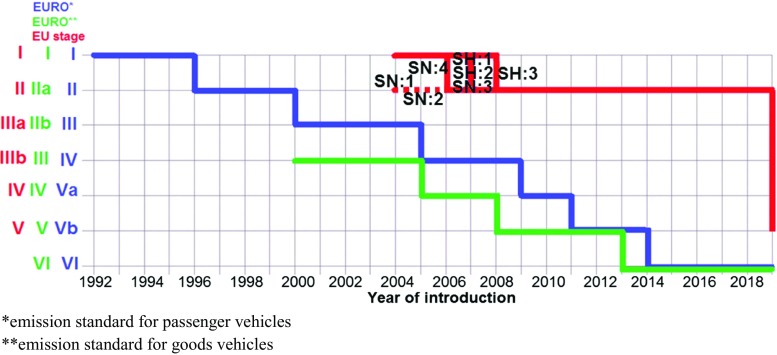
Implementation and revision of the European standards of the emission limits of toxic compounds in exhaust gases for spark ignition engines: passenger vehicles Euro*, goods vehicles Euro**, non-road mobile machinery Stage

**Fig. 3:**
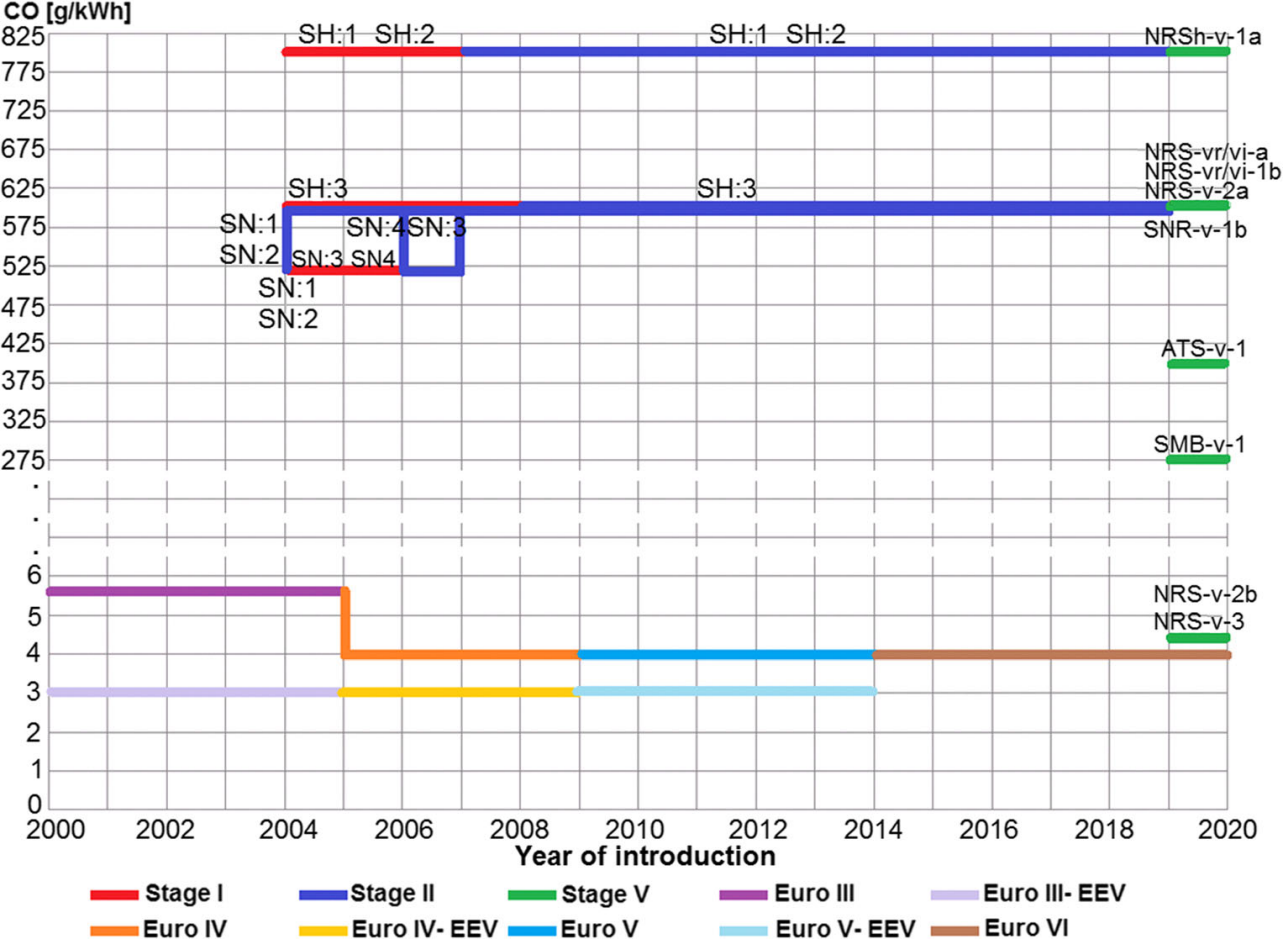
Allowable emission limits of carbon monoxide CO in exhaust gases of spark ignition engines formulated in standards Euro and Stage

**Fig. 4:**
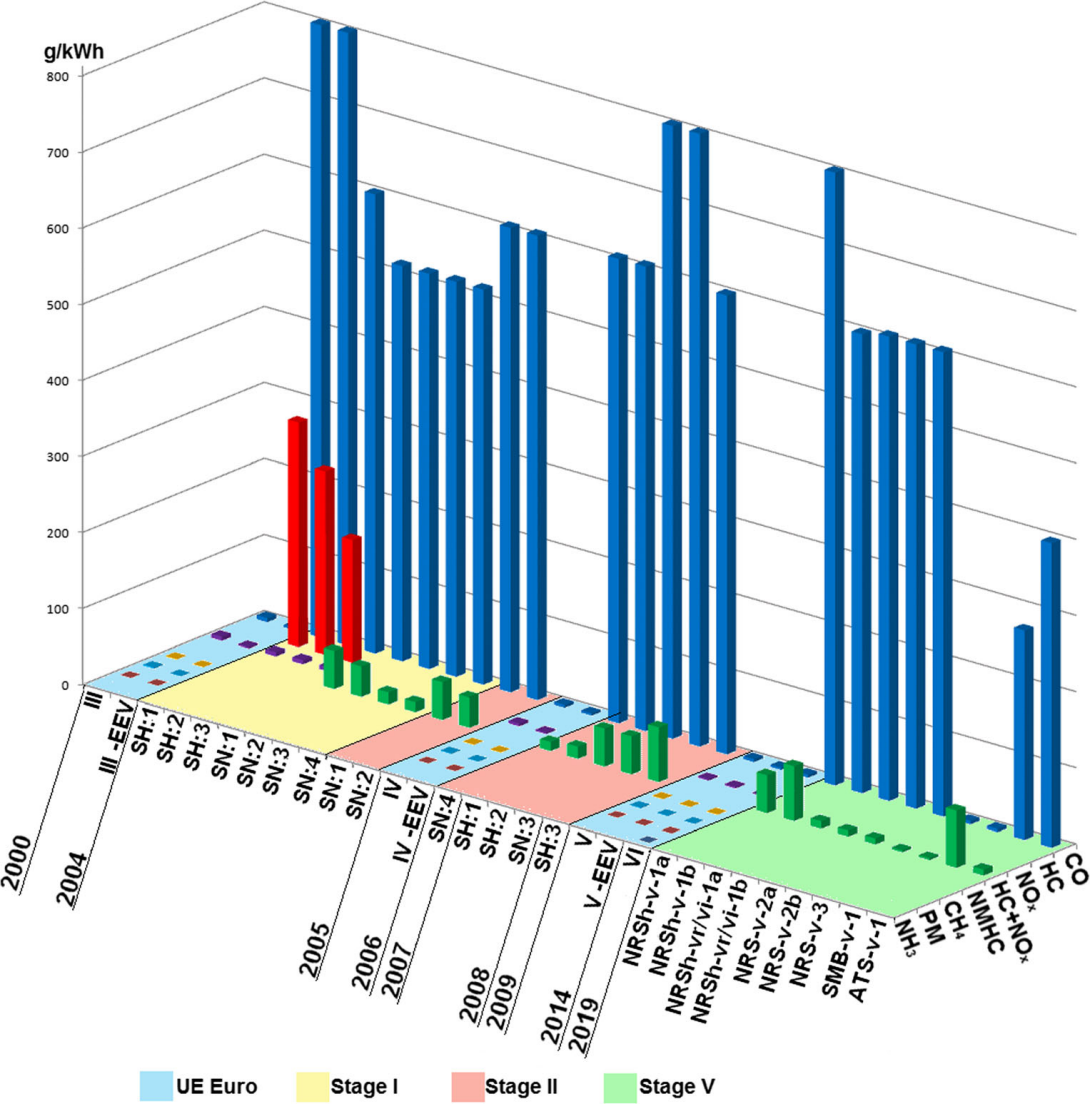
Allowable emission limits of harmful compounds in exhaust gases of spark ignition engines formulated in standards Euro and Stage

The allowable European emission limits of harmful exhaust gases for passenger vehicles and goods vehicles with the allowable vehicle mass higher than 2610 kg and busses with total mass higher than 5 t and delivery trucks with total mass higher than 12 t—irrespective of their rated masses—are measured in units g/kWh (Table 9). The values of the emission limits measured in this unit allow for their comparison with the values of the emission limits for non-road mobile machinery (Figs. 2, 3, 4). The review of goods vehicles was restricted to the emission limits for spark ignition engines. Euro 1 and Euro 2 standards for such type of vehicles are not related to spark ignition engines. The presented emission limits in Table 1 are also obligatory for compression ignition engines during ETC test in transient conditions (Delphi-Worldwide Emissions Standards, 2017; Gołębiowski et al. 2013). Table 9 presents the allowable emission limits which are divided according to the standards, and it shows the emission limits for enhanced environmental friendly vehicles (EEV). The EEV standard extorts extremely low emission level of particulate pollutants which are serious hazard for health and quality of life (Table 11).

**Table 11:** Allowable European emission limits of toxic compounds in exhaust gases for passenger and goods vehicles with the allowable weight higher than 2610 kg and busses with total weight higher than 5 t and delivery trucks with total weight higher than 12 t regardless of their rated mass and equipped with spark ignition engine and date of implementation (Delphi-Worldwide Emissions Standards, 2017; Gołębiowski et al. 2013; Council Directive, 1991, 1993; Directive, 1994/12/WE; Directive, 199669/WE; Directive, 1998/69/WE; Directive, 2002a/80/WE; Regulation 2007/715/WE)

EURO	Date	Carbon monoxide (CO)	Ammonia (NH_3_)	Nitrogen oxides (NO_x_)	Non-metallic hydrocarbons (NMHC)	Methane (CH_4_)^2^	Solid particles (PM)
		(g/kWh)					
III	2000	5.45	–	5.0	0.78	1.6	0.16/0.21^1,3^
III-EEV	2000	3.0	–	2.0	0.4	0.65	0.02^3^
IV	2005	4.0	–	3.5	0.55	1.1	0.03^3^
IV-EEV	2005	3.0	–	2.0	0.4	0.65	0.02^3^
V	2009	4.0	–	2.0	0.55	1.1	0.03^3^
V-EEV	2009	3.0	–	2.0	0.4	0.65	0.02^3^
VI	2014	4.0	0.01	0.46	0.5	0.5	0.01

^1^For engines with engine cubic capacity below 0.75 dm^3^ for one cylinder and the power rating > 3000 min^−1^

^2^In scope of standards Euro 3–Euro 5, this limit is related with the engines which are supplied with natural gas (NG), but in scope of standard Euro 6 this limit is related with spark ignition engines

^3^In scope of standards Euro 3–Euro 5, this limit is not related with the engines which are supplied with gas

## Conclusions

The main aim of this review is the presentation of the actual state of the art on the subject of the influence of the EU regulations on the global emission of contamination from non-road machinery. Their impact on the air quality cannot be omitted during consideration and analysis of all pollutant factors. On the basis of detailed analysis of literature, the authors of this paper have concluded that currently available information on the influence of non-road machinery and other devices in households driven by liquid fuels is insufficient. A lack of analysis of the influence of these machines on the general level of contamination and a lack of the regulations will be the reason of climate warming and larger smog formation.

The directives related to the emission limits of harmful compounds in exhaust gases for non-road mobile machinery with spark ignition engine are more liberal than the directives related to passenger or goods vehicles. Some legal activities are made for tightening the regulations for this machinery, and homologation limits are implemented with a few years of delay in relation to road vehicles. The values of the emission limit of harmful compounds in exhaust gases for this machinery are still considerably higher than for road vehicles. This fact has an influence on construction and control processes of these engines. In group of these engines the fuel supply system is made in majority with the application of the carburetor. The control possibilities of this system are considerably limited and therefore just a few percentage of passenger vehicles produced in 1993 was equipped with this type of fuel supply system (Wendeker 1999). One should expect that the innovations from road vehicle engines will be implemented into the engines for propulsion of non-road mobile machinery. The leading manufacturers, who implement the innovations into this group of engines e.g. Kohler Engines, Briggs & Stratton, Honda, Kawasaki, Subaru Robin, Yamaha (Briggs & Stratton gasoline engines, 2017; Honda gasoline engines, 2017; Kawasaki gasoline engines, 2017, Kohler Power Group gasoline engines, 2017; Subaru Industrial Power Products gasoline engines, 2017; Yamaha gasoline engines, 2017), present the innovative solution of the engine with the control process which is based on the integrated injection-ignition systems where the injection is done into the suction manifold. These systems are free from catalytic reactor and feedback in control process. Integrated electronic injection-ignition systems are characterized with better control of fuel dosage in comparison with carburetors. However, these engines are in the minority on the market of offered engines due to the liberal emission rules for non-road mobile machinery. The standards for passenger vehicles are more rigorous, but due to the measured units, we cannot compare directly these ones with the standards for non-road mobile machinery. The standard for passenger vehicles is more and more liberal in relation to allowable weight of the vehicle. Only standards for goods vehicles are represented in the unit which allows for comparison, and their emission limits are more rigorous than for non-road mobile machinery.

In case of engines for hand-held devices, the emission limits of toxic compounds in exhaust gases are the most liberal, but the most important in these devices are compact construction, weight, and generated vibration. The manufactures of these devices: Makita, Stihl and Shindaiwa (Makita hybrid of two- and four-stroke engines, 2017; Shindaiwa hybrid of two- andfour-stroke engines, 2017; Stihl hybrid of two- and four-stroke engines, 2017) propose the innovative engines which are the hybrid of two- and four-stroke engines. These ones are characterized with the power increase, reduction of toxic compounds emission, and reduction of noise. The fuel supply system of this engine is still based on the carburetor.

It was shown that the legal requirements impose the level of innovation of construction of ignition-injection systems. The local level of pollutants emission depends on the level of innovation of applied systems—this fact has a special meaning in urban agglomerations. This situation will lead to searching for the emission limits of harmful compounds in exhaust gases in all branches of the industry—including propulsions of machines and devices for non-road working machines. However, the main stimulation for searching will be the implementation of more rigorous standards of the emission limits which will be close to the emission limits for road vehicles. To restrict the emission level, one should start the works on the implementation of operation control systems of road vehicle engines into non-road mobile machinery engines. The application of homologation standards and innovative solutions transferred from automotive vehicles will allow to restrict the global emission of harmful compounds in exhaust gases.
